# EDEL: enhancing dense retrievers for curation of biomedical knowledge bases

**DOI:** 10.1093/bioinformatics/btag490

**Published:** 2026-07-02

**Authors:** Xing David Wang, Ulf Leser

**Affiliations:** Department of Computer Science, Humboldt-Universität zu Berlin, Berlin, 10099, Germany; Department of Computer Science, Humboldt-Universität zu Berlin, Berlin, 10099, Germany

## Abstract

**Motivation:**

Retrieval of relevant papers from the literature is the first step in curating high-quality biomedical knowledge bases. While BM25 has long been the method of choice, dense retrieval models achieved improved accuracy by embedding queries and documents into dense vector representations. Existing knowledge bases provide a natural source for deriving query-document pairs for training such models. Current training approaches, however, do not take into account that some evidence described by knowledge base entries may only be partially expressed in document abstracts, while the full evidence is often contained in inaccessible full texts, introducing noise into binary relevance labels. In addition, existing approaches only make limited use of the knowledge base structure for selecting negative samples during training.

**Results:**

We propose EDEL, a novel dense bi-encoder for biomedical knowledge base curation to enable curators to find relevant papers for annotation faster. It introduces a loss function using graded relevance scores instead of binary labels to facilitate learning from partially grounded examples, together with a structured sampling strategy that exposes the model to diverse and hard negative examples during training. We evaluate EDEL’s performance in two curation settings, namely precision oncology (on CIViC and OncoKB) and post-translational modifications (on UniProt). EDEL outperforms other state-of-the-art models in NDCG@10 by 1.5 and 3.4 percentage points, respectively. Ablation studies show the effectiveness of both innovations. These results indicate that EDEL can substantially improve literature retrieval for biomedical knowledge base curation.

**Availability and implementation:**

Code to reproduce our results is available at: https://github.com/WangXII/edel_repo.

## 1 Introduction

Integrating knowledge from the rapidly growing biomedical literature into curated knowledge bases (KBs) is essential for many fields of biomedical research, yet it remains a labor-intensive and time-consuming task (Rieke *et al.* 2022). In precision oncology, for example, resources such as CIViC (Griffith *et al.* 2017) and OncoKB (Chakravarty *et al.* 2017) link cancer-associated genomic variants to potential treatments, but their continuous updating depends on manual expert review (Rodriguez-Esteban 2015, Poux *et al.* 2017). The first step in the curation process is the retrieval of the most relevant works from thousands of new publications each year. Performing this step as accurately as possible is indispensable to maximize a curator’s efficiency and thereby helping to scale the curation process (Lee *et al.* 2016, Poux *et al.* 2017, Lever *et al.* 2019).

The goal in information retrieval is to provide a ranked list of documents (e.g. PubMed abstracts) according to their relevance to a given query. While BM25 and its variants were the method of choice for this step, recent research in information retrieval has shown that dense retrieval models, when properly trained, rank documents with considerable higher accuracy (Hofstätter *et al.* 2021, Santhanam *et al.* 2022, Jin *et al.* 2023). Dense retrieval models embed queries and documents independently into numeric vectors and use similarity between these representations for ranking (Karpukhin *et al.* 2020). However, these models require large volumes of training data, which is not always available. Existing strategies to obtain such training data face important limitations. Manual annotation by domain experts yields query-document pairs with high quality (Tsatsaronis *et al.* 2015) but is costly and difficult to scale. Search engine logs, by contrast, provide large quantities of implicit relevance signals (Rekabsaz *et al.* 2021, Jin *et al.* 2023), yet these signals are often noisy and only loosely reflect true relevance.

Structured knowledge bases can serve as a third source of supervision signals for retrieval models. A KB entry typically consists of a set of entities together with links to supporting literature. For example, in a precision oncology KB such as CIViC, genes and variants can define a query, while treatments or clinical outcomes can define a curation target. The linked PubMed article then serves as the relevant document.

However, standard training recipes applied to query-document pairs derived from biomedical KBs suffer from two major limitations. The first issue arises because the facts represented by a KB entry are often only partially expressed in linked abstracts and only appear completely in the full texts (which are often not openly accessible; full text articles licensed for automatic text mining currently make up only 7.5 million of the 37 million articles currently indexed in PubMed.). For instance, we can only fully match about 30% of CIViC entries in abstracts (with gene, variant, and treatment), even when using extensive synonym lists. As a result, entities from KB entries are frequently missing from accessible abstracts, introducing noisy training signals (Mintz *et al.* 2009, Surdeanu *et al.* 2012). The alternative, to only include fully mappable KB entries during training data construction, however, heavily reduces the size of the available training data.

The second limitation is that current negative sampling methods do not exploit the structured nature of KBs. As all KB entries share the same set of entity types, potential negative samples from other entries can be stratified according to their entity overlap with a positive KB entry, allowing the explicit sampling of hard negatives. For example, an entry sharing the same gene but a different variant constitutes a harder negative than an entry without any overlapping entities. Instead, current approaches rely on random sampling, in-batch sampling (Karpukhin *et al.* 2020), iteratively updated models or knowledge distillation from larger teacher models (Xiong *et al.* 2020, Hofstätter *et al.* 2021). The former suffers from the low quality of the resulting negative instances with random samples often being “too easy” to classify and leading to weak training signals (Xiong *et al.* 2020), while the latter is computationally more expensive requiring the predictions of a much bigger teacher (Hofstätter *et al.* 2021, Santhanam *et al.* 2022).

**Figure 1:**
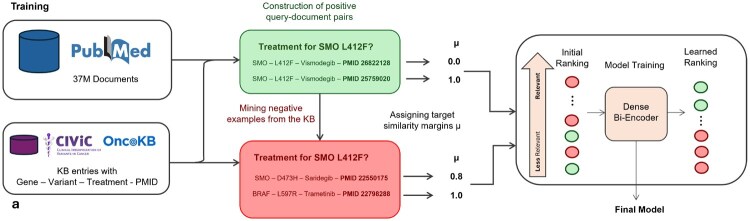
Overview of the EDEL framework. (a) Model training relies on literature abstracts as reference documents whereas the training data for query-document associations comes from biomedical knowledge bases such as CIViC and OncoKB. Using a layered loss function with noisy positives as well as explicitly sampling negative examples from other KB entries, we train a dense bi-encoder for ranking document relevance.

In this study, we present EDEL, a novel dense retrieval framework designed to accelerate the curation of biomedical knowledge bases by introducing two key enhancements (see Fig. 1 for an overview): First, EDEL addresses partially grounded KB entries by introducing a layered margin loss that reflects partially grounded evidence in document abstracts. Instead of discarding partially matched entries, EDEL assigns them lower relevance than fully grounded examples, allowing such entries to remain useful, positive training samples. Second, we ensure a high quality, diverse set of negatives by carefully choosing both easy (i.e. trivial) as well as hard negatives. We achieve this by stratifying KB entries according to their entity overlap with a given positive example and explicitly sampling negatives from each group.

We evaluate EDEL in two biomedical fields that highly depend on curated KBs. For precision oncology, we consider the KBs CIViC (Griffith *et al.* 2017) and OncoKB (Chakravarty *et al.* 2017), both of which are commonly used for interpretation of genomic variants in cancer patients (Rieke *et al.* 2018, Tamborero *et al.* 2022). Second, we consider the curation of post-translational protein modifications in UniProt Consortium (2019), where the task is to retrieve known catalysts for a modified protein site. EDEL outperforms several state-of-the-art baselines in both fields by up to 1.5 and 3.4 percentage points (pp) in NDCG@10, respectively. Specifically, it increases retrieval accuracy in identifying the very same publications that curators have annotated as relevant in the knowledge base, ensuring more precise search results. Second, it also reliably retrieves additional relevant documents that were not originally annotated as gold references, thereby reducing the risk of missing potentially relevant evidence. Together, these improvements enable more precise literature retrieval, supporting more efficient and comprehensive knowledge base curation.

## 2 Methods

### 2.1 Mining structured KBs for query-document pairs

We describe how structured KBs can be leveraged to mine query-document pairs for retrieval. We assume that each KB stores their data in tabular form, where each row (or entry) represents one biomedical relation. The columns contain the entities participating in that relation (e.g. genes, variants and drugs) together with links to one or more external sources supporting that relation (e.g. a PubMed ID). In our KB curation setting, we divide the entities into query entities and answer entities, where the latter correspond to the curation targets. We define the retrieval task as finding documents supporting the relation between the query entities and potentially new answer entities.

For each KB entry, a natural language query can be constructed by filling the query entities into a predefined template and specifying the type of the desired answer entities. For example, in our precision oncology curation task, we might fill the template *Treatment for gene* $e_{1}$  *and variant* $e_{2}$*?* with the gene *KRAS* and variant *G12D*. The literature source linked in the entry, usually through the PubMed ID, serves as the target document to retrieve.

### 2.2 Datasets

To train and evaluate retrieval models in our KB curation setting, we mined query-document pairs from two different KBs leaving us with two new datasets.

We derived the first dataset, Precision Oncology (PO), from the two precision oncology KBs CIViC (Griffith *et al.* 2017) and OncoKB (Chakravarty *et al.* 2017). As described, the queries consist of gene and variant entities and potential treatments targeting the genetic alteration form the answer entities here. Our final PO dataset contains around 400 unique genes, 1060 unique gene-variant queries and 3260 positive query–document pairs (see Table 1).

**Table 1:** Number of unique gene/protein entities, queries, positive query-document pairs and entity tuples extracted from the two precision oncology (PO) KBs CIViC, OncoKB and the UniProt KB for post-translational modifications (PTM).

	**Precision oncology**			PTM
	CIViC	OncoKB	Combined	UniProt
Genes/Proteins $e_{1}$	333	158	395	2002
Queries $q=(e_{1},\ldots )$	939	164	1064	2455
Query-answer pairs $\left(e_{1},\ldots ,e_{n}\right)$	2262	458	2666	2861
Positive query-doc pairs (q,d)	2126	1187	3261	4492

**Table 2:** Margin classes, their margin values μ and number of positive and negative examples for the two datasets Precision Oncology and Post-Translational Modifications.

**Margin ** μ	**Precision oncology**		**PTM**	
	Positives	Negatives	Positives	Negatives
0.0	827	0	563	0
0.2	2065	16 775	1179	2015
0.6	218	9912	1405	1260
0.8	0	149 083	1294	45 736
1.0	1117	176 079	51	43 473
1.2	200	0	0	37 980
Total	4427	351 849	4492	130 464

We derived the second dataset, Post-Translational Modifications (PTM), from the UniProt KB. Our curation targets catalyst entities for given queries consisting of a protein, its modified residue site, and the PTM type. An exemplary query in the PTM dataset is *Catalysts for the phosphorylation of AKT1 at serine 473?*, where *phosphorylation* is the PTM type, *AKT1* is the *modified* protein, and *serine 473* is the modified Residue.

Both datasets share the same underlying PubMed corpora for their documents, containing around 36 million documents published until 2024. A major challenge in biomedical IR is that only a subset of full-text documents are openly accessible (about 25% as of October 2025). Although abstracts are available via PubMed, they often do not contain all the entities given in a KB entry. In our datasets, only about 20% of samples in PO and about 12.5% in PTM have fully covered entities in their corresponding abstracts. More details about the used datasets and the PubMed corpus can be found in Supplementary Section A.

### 2.3 The EDEL framework

We present EDEL, a new framework to efficiently leverage structured knowledge bases (KBs) for training dense retriever models. To achieve this, EDEL introduces two key innovations: Given query-document associations mined from a KB, (i) EDEL uses a layered, contrastive loss function to handle graded relevance levels from partially grounded positives (which we call noisy positives), and (ii) EDEL carefully selects negatives by balanced sampling from both hard and easy negatives.

As for the model architecture, EDEL uses a shared BERT encoder for embedding both queries and documents. We initialize the encoder from the BioLinkBERT-base checkpoint by Yasunaga *et al.* (2022) consisting of 110 M model parameters. We split the KB entries into a separate train split, development split and test split. We fine-tune separate retriever models on each KB task, one on the PO dataset and one on the PTM dataset.

#### 2.3.1 Noisy positives and layered margin loss

A major challenge in biomedical IR is that many referenced full texts are not openly accessible, while abstracts often mention only a subset of the entities contained in a KB entry. Only about 20% of all entries in the PO dataset and 12.5% in the PTM dataset contain every entity in their linked abstract.

Therefore, from all KB-derived positive samples, we distinguish between completely grounded positives (complete positives CP), where all entities can be matched in the linked abstract, and partially grounded positives (noisy positives NP), where only a subset of entities can be matched. We define an entity as matched if either the entity itself or one of its synonyms is mentioned in the linked abstract. Rather than discarding the noisy positives entirely, we assign them different relevance margins depending on the number of matching entities. Examples with the same pattern of matched entity types (e.g. genes and variants matched, treatments not) are grouped into the same margin class.

To measure relevance between queries and documents, we use cosine distance as the scoring function. Its bounded range between 0 and 2 allows us to define class-specific relevance margins, where smaller distances indicate more relevant query-document pairs:


(1) $$\text{dist}(q,d)=1-\frac{\vec{v}(q)\cdot \vec{v}(d)}{\left|\vec{v}(q)|\;|\vec{v}(d)\right|},$$

where $\vec{v}(q)$ and $\vec{v}(d)$ are the embeddings of the special [CLS] token in BERT-style models representing the vector for query *q* and document *d*, · denotes the scalar product and $\left|\vec{v}(q)\right|$ the vector norm.

For the PO dataset, we distinguish five positive margin classes, including one class of complete positives and four classes of noisy positives (see Table 3). We assign margin values ranging between 0 and 2 to each class as part of hyperparameter optimization, assigning larger values to classes with fewer matching entities, indicating less reliable positive evidence. For instance, consider the KB entry *(SMO, L412F, Vismodegib, PubMed ID 26822128)* from Fig. 1. As all entities are mentioned in that example abstract, we consider it a complete positive and assign a minimum margin value of 0.0 to the corresponding query-document pair.

**Table 3:** Margin classes for positive samples on the Precision Oncology dataset.

Class	μ	EntityInText			
		Gene	Variant	Treatment	Count
1	0	True	True	True	827
2	0.2	Either one matching		True	2065
3	0.6	True	True	False	218
4	1.0	Exactly one entity matching			1117
5	1.2	False	False	False	200

We check whether each KB entity is mentioned in its linked abstract by using True and False. If True, the entity is mentioned; if False, the entity is not mentioned.

To learn to distinguish the different classes and their margin values, we extend the classic contrastive loss function by Hadsell *et al.* (2006) to a graded relevance version:


$$\begin{array}{c} \ell (q,d)=y(d)\cdot \max{\left(0,\text{dist}(q,d)-\mu^{\prime }(d)\right)}^{2}\\ +(1-y(d))\cdot \max{\left(0,\mu^{\prime }(d)-\text{dist}(q,d)\right)}^{2}. \end{array}$$

where the label y(d)=1 for positive sample documents *d* and y(d)=0 for negative ones, and $\mu^{\prime }(d)=\arccos(1-\mu (d))$ denotes the angular value corresponding to the class-dependent margin μ(d) of document *d*. This encourages closer alignment between queries and relevant documents (with smaller margins) and separation from less relevant or negative ones (with larger margins). Table 2 provides an overview of the margin values and classes used in both datasets. Table 3 provides a detailed list of margin values μ as well as the matching entities for each of those classes on the PO dataset, Table S1 provides the corresponding values on the PTM dataset and Supplementary Section E provides details on hyperparameter optimization.

#### 2.3.2 Hard negatives sampling

EDEL leverages the structured nature of KBs to select informative negative examples from other KB entries. We quantify overlap between two KB entries by counting the entities they share. Negative examples sharing more entities with a positive entry are considered harder negatives than examples without overlapping entities. For instance, in the PO dataset, the negative entry (*SMO, D473H, Saridegib*) shares the gene entity with the positive entry (*SMO, L412F, Vismodegib*) and therefore forms a harder negative than an unrelated entry such as (*KRAS, G12C, Sotorasib*).

For answer entities, we consider any non-empty answer entity as overlapping, since a query may have multiple valid answers. For example, the entry (*SMO, L412F, -*) overlaps in the gene and variant entities but not in the treatment entity.

To obtain more reliable supervision signals, we filter the negative candidate pool for KB entries where all entities are explicitly mentioned in the linked abstract.

As for positive samples, negatives with the same overlap pattern are stratified into the same margin class. For each positive example, we sample up to a maximum m=50 negatives from each margin class. We assign smaller margins to negatives with larger entity overlap, reflecting their greater similarity to positive samples. Initial margin values are determined heuristically from the number of overlapping entities and further tuned during hyperparameter optimization (see Supplementary Section E for more details).

In addition to these KB-derived negatives, we include two more, external sources for negative mining: (i) BM25 negatives by retrieving documents containing the same gene/protein entity but not containing any trigger words related to the answer entity type (for instance, *therapy, treatment, drug* on the PO task, see Supplementary Section D) and (ii) random PubMed negatives.

Based on the different numbers of query and answer entities in both datasets, we end up distinguishing four negative classes in the PO dataset (see Table 4) and five in the PTM dataset (see Table S4). Table 4 provides a full overview of all our negative margin classes and their corresponding values on the PO dataset (see Tables S3 and S4 for the corresponding overview on the PTM dataset).

**Table 4:** Margin classes for negative samples on the Precision Oncology dataset.

Class	μ	SameGene	SameVariant	AnyTreatment	InText	Count
1	0.2	True	False	True	Variant=False	16 775
2	0.6	True	False	True	All=True	9769
		True	True	False	All=True	143
3	0.8	True	False	False	All=True	3745
		False	–	True	All=True	88 078
		BM25 negatives of same gene			–	57 260
4	1.0	False	–	False	All=True	87 544
		Random PubMed negatives			–	88 535

We explicitly check whether entities of another KB entry overlap with those of a positive sample. We also check that all KB entities are mentioned in their linked abstracts. Important to note is that Class 1 is harder because the wrong variant is *not* mentioned while Class 2 is easier because the wrong variant is *explicitly* mentioned.

Closest to our negative sampling approach are margin-based distillation methods like TAS-B (Hofstätter *et al.* 2021) which balance negative selection by sampling from the margin distribution of a teacher model. In contrast, EDEL derives such soft relevance signals directly from KB structure and therefore does not require a teacher model.

### 2.4 Evaluation

#### 2.4.1 Baselines

To examine how well our two proposed KB retrieval tasks can be solved without any domain-specific fine-tuning, we compare our EDEL approach to three strong baselines in a zero-shot setting: BM25 (Robertson and Zaragoza 2009), a sparse vector space model based on lexical features, remains a strong baseline for first-stage retrieval (Thakur *et al.* 2021, Rajapakse *et al.* 2024). MedCPT (Jin *et al.* 2023) is a dense bi-encoder trained on over 255 million PubMed click logs from PubMed users. ColBERTv2 (Santhanam *et al.* 2022) is a neural retriever that uses separate embeddings for each token in a document instead of a single document embedding. It employs a late interaction mechanism to combine the individual token embeddings from the query and the document at inference time.

Secondly, we compare EDEL to two additional fine-tuned dense retrieval model to examine the effectiveness of our proposed training method over standard fine-tuning. For this, we further fine-tune the MedCPT retriever and ColBERTv2 on complete positives and in-batch negatives plus BM25/random negatives (see Section 4.1 for discussion on fine-tuning details and Supplementary Section D on details about BM25 negatives).

#### 2.4.2 Evaluation metrics

Following popular retrieval benchmarks like BEIR (Thakur *et al.* 2021) and MTEB (Muennighoff *et al.* 2023), we report the NDCG (normalized discounted cumulative gain) at cutoff threshold k=10 and k=50, that means, considering only the top-*k* retrieved documents from the answer ranking. NDCG ranges from 0 to 1 with 1 being an perfect ranking having all relevant documents in the top-*k* search results.

In order to measure our ability to retrieve gold answer entities (our NDCG score does not distinguish between noisy and complete positives, only whether a gold document is found), we introduce the Entity Recall metric and evaluate it at k=10 and k=50. We count an answer entity as successfully found under the Entity Recall metric if (i) there is at least one mention of the answer or one of its synonyms in any of the top-k retrieved documents and (ii) there is at least one mention of the corresponding gene/protein of interest in the document, that is, indicating that the relation is likely contained there.

## 3 Results

Table 5 reports the retrieval results on the PO and PTM datasets. EDEL consistently outperforms all zero-shot baselines across both tasks and evaluation metrics. On PO, EDEL improves NDCG@10 from 11.17% for BM25 and 10.95% for ColBERTv2 to 18.62%. Similar improvements are observed on PTM, where EDEL achieves 24.24% in NDCG@10 compared to 20.50% for BM25 and 18.59% for ColBERTv2. These results indicate that EDEL more effectively ranks relevant biomedical abstracts under KB-derived supervision.

**Table 5:** Retrieval performances on the Precision Oncology and Post-translational modifications datasets.

**Dataset** (→)	**Precision oncology**						**Post-translational modifications**					
	NDCG		MAP		Entity Recall		NDCG		MAP		Entity Recall	
**Model Name** (↓)	@10	@50	@10	@50	@10	@50	@10	@50	@10	@50	@10	@50
**Zero-shot models**												
BM25	11.17	12.37	9.07	9.29	21.21	43.56	20.50	23.70	16.70	17.47	42.11	55.73
ColBERTv2	10.95	12.46	9.03	9.32	31.82	53.79	18.59	21.60	14.78	15.49	39.96	50.54
MedCPT	9.15	11.18	7.57	7.89	27.27	48.48	13.10	17.30	10.07	11.05	35.13	48.39
**Models fine-tuned on KB entries**												
ColBERTv2	17.12	18.46	**14.39**	14.68	39.39	57.58	20.80	23.41	16.94	17.55	44.09	55.20
MedCPT	12.91	15.46	10.87	11.34	28.41	53.79	19.64	23.23	15.73	16.54	40.14	57.53
EDEL	**18.62**	**23.66**	14.07	**15.24**	**53.41**	**73.48**	**24.24**	**26.47**	**19.73**	**20.21**	**50.72**	**61.65**

Results are reported for zero-shot baselines and models fine-tuned on KB-derived supervision. Best score in each column is marked in bold, the second best is underlined.

Fine-tuning existing dense retrievers on KB-derived supervision substantially improves performance over their zero-shot counterparts. Fine-tuned ColBERTv2 reaches 17.12% in NDCG@10 on PO and 20.80% on PTM, confirming that the datasets provide meaningful retrieval supervision. Nevertheless, EDEL consistently outperforms these fine-tuned baselines, improving NDCG@10 by 1.5 percentage points (pp) on PO and 3.4 pp on PTM over fine-tuned ColBERTv2. EDEL also achieves substantially higher Entity Recall@10 values, with gains exceeding 14 pp on PO and 6.7 pp on PTM. This suggests that EDEL’s graded relevance modeling and structured negative sampling strategy provide additional benefits beyond standard in-batch fine-tuning.

The gains remain consistent at larger cutoff ranks. At cutoff rank 50, EDEL continues to outperform all baselines across both datasets and metrics, with the largest improvement observed on PO where NDCG@50 increases to 23.66% compared to 12.46% for zero-shot ColBERTv2 and 18.46% for fine-tuned ColBERTv2.

EDEL also achieves substantially higher Entity Recall values than competing methods, suggesting that the model retrieves biologically relevant documents even when they are not annotated as gold documents in the KB. To assess this, we manually inspected the top-3 retrieved documents for ten test queries in the PO dataset for which none of the top retrieved documents had been curated in the KB. Among the 30 retrieved abstracts, 13 appeared relevant (discussing a treatment for the same variant), 14 were potentially relevant but required full-text verification (discussing a treatment for the same gene but not specific to any variant), and only 3 appeared irrelevant. This indicates that EDEL frequently retrieves semantically related evidence beyond the curated annotations.

## 4 Discussion

### 4.1 Fine-tuning ColBERT

To ensure that our gains are not due to under-tuned baselines, we carefully fine-tuned ColBERTv2 under several training configurations. We distinguish between training on KB-derived positives for which all relevant entities can be matched in the linked abstract, complete positives (CP), and on partially grounded entries, noisy positives (NP). We additionally include externally mined negatives (ExtNeg) derived from BM25 and other random PubMed documents, alongside standard in-batch negatives. We conducted a ColBERTv2 fine-tuning ablation under these configurations and report the results in Table 6.

**Table 6:** Fine-tuning the ColBERTv2 model on our KB datasets.

**Dataset (** → **)**	**Precision oncology**						**Post-translational modifications**					
	NDCG		MAP		Entity Recall		NDCG		MAP		Entity Recall	
**Model Name (** ↓ **)**	@10	@50	@10	@50	@10	@50	@10	@50	@10	@50	@10	@50
ColBERTv2	10.95	12.46	9.03	9.32	31.82	53.79	18.59	21.60	14.78	15.49	39.96	50.54
+NP,+CP,+BatchNeg	12.93	13.85	10.27	10.56	20.45	39.02	12.77	15.07	10.58	11.09	27.96	41.04
+CP,+BatchNeg	15.68	17.45	13.21	13.65	37.12	53.02	20.14	22.95	16.50	17.10	43.19	53.76
+CP,+BatchNeg,+ExtNeg	**17.12**	**18.46**	**14.39**	**14.68**	**39.39**	**57.58**	**20.80**	**23.41**	**16.94**	**17.55**	**44.09**	**55.20**

CP denotes complete positives, NP noisy positives, BatchNeg in-batch negatives and ExtNeg externally mined negatives such as from BM25 and random PubMed documents. Best score in each column is marked in bold.

We show that adding noisy positives (+NP,+CP,+BatchNeg) and treating them as complete positives reduces retrieval performance compared to using complete positives only (+CP,+BatchNeg). This holds true across both PO and PTM datasets across all metrics. In addition, adding external negative sources such as from BM25 or other PubMed documents proves to be a simple way to improve baseline retrieval performance.

### 4.2 EDEL ablation studies

In the EDEL ablation studies reported in Table 7, we examine the contributions of each individual component of our proposed EDEL model: graded relevance modeling using the layered loss function (LL), the inclusion of noisy positives (NP), and structured negative sampling using hard negative classes (HNeg) and external negatives (ExtNeg).

**Table 7:** Ablation studies on the EDEL model.

**Dataset** (→)	**Precision Oncology**						**Post-translational Modifications**						Notes
	NDCG		MAP		Entity Recall		NDCG		MAP		Entity Recall		
**Model Name (** ↓ **)**	@10	@50	@10	@50	@10	@50	@10	@50	@10	@50	@10	@50	
EDEL	**18.62**	**23.66**	14.07	15.24	**53.41**	**73.48**	24.24	26.47	19.73	20.21	**50.72**	61.65	
-ExtNeg	17.09	20.42	**14.94**	**15.76**	46.21	66.29	20.27	23.03	15.77	16.36	44.44	60.39	
-ExtNeg,-HNeg	16.19	18.54	12.97	13.54	30.68	53.79	**26.48**	**30.02**	**22.51**	**23.34**	44.44	**62.01**	+BatchNeg instead
-LL	17.77	21.36	14.65	15.53	40.91	61.36	22.64	25.59	18.60	19.30	43.73	57.53	
-NP	14.42	16.06	11.82	12.16	46.97	67.42	17.26	21.43	13.38	14.31	38.89	51.08	
-LL,-NP	16.28	17.09	13.00	13.20	30.30	40.91	24.49	27.24	20.47	21.12	39.96	51.61	
-LL,-NP,-ExtNeg,-HNeg	14.41	16.83	12.48	13.01	23.11	45.45	8.43	11.69	5.95	6.65	28.67	43.19	+BatchNeg instead

Each row removes one component from the full EDEL setup unless otherwise noted. LL stands for layered loss function, NP for noisy positives, ExtNeg encompasses external negatives from BM25 and random PubMed documents, HNeg denotes hard negative mined from groups of stratified KB entries. Rows marked “+BatchNeg instead” replace the removed negative sources with standard in-batch negatives. Numbers of the best performing model in each column are marked in bold, the second best are underlined.

As expected, training EDEL without any of our proposed components (-LL,-NP,-ExtNeg,-HNeg) but only in-batch negatives performs the worst across all ablation studies. This drop is particularly pronounced on UniProt, where the model loses 7.8 pp in NDCG@10 and 10.2 pp in Entity Recall@10 relative to the next-worst ablation. This suggests that effective retrieval training on UniProt depends strongly on informative negative examples, particularly when fine-tuning from a general-purpose encoder as BioLink-BERT.

Removing the layered loss function (-LL) generally reduces retrieval performance, especially for NDCG and Entity Recall although the MAP scores in the PO dataset slightly increase. This suggests that the layered loss stabilizes training with noisy positive samples. In contrast to the fine-tuned ColBERTv2 baseline, we show that removing noisy positives (-NP) substantially reduces retrieval performance. All examined retrieval metrics drop across both tasks, the NDCG@10 by 4.2 pp on PO and 6.5 pp on PTM, the Entity Recall@10 scores by 6.4 pp on PO and 11.8 pp on PTM. Together, these results support the use of graded relevance rather than binary labels for KB-derived supervision.

Looking at the removal of external negatives (-ExtNeg) and hard negatives (-ExtNeg, -HNeg), the results show a more nuanced pattern. On the PO dataset, removing external negatives and hard negative classes generally reduces NDCG and Entity Recall, suggesting that structured negative sampling improves retrieval. On PTM, however, replacing hard negatives and external negatives with in-batch negatives reduces Entity Recall, while improving NDCG and MAP retrieval scores.

We suspect this behavior is caused less by the negative sampling strategy itself than by suboptimal margin assignments arising from the larger joint hyperparameter space introduced by noisy positives and hard negatives. Supporting this interpretation, the ablation retaining hard negatives while removing layered loss and noisy positives (-LL,-NP) still achieves strong NDCG and MAP scores on PTM. More effective optimization of the margin hyperparameters beyond our grid-search approach (see Supplementary Section E) may therefore further improve and stabilize performance.

## 5 Conclusion

Keeping knowledge bases up-to-date as the biomedical literature expands is a challenging task yet of much practical relevance. In this study, we have proposed EDEL, a novel dense retriever that leverages the structure of KB records to sample informative, hard negatives and applies a layered contrastive loss function to model varying degrees of document relevance. Our experiments demonstrate that EDEL outperforms both zero-shot and fine-tuned baselines, highlighting the effectiveness of combining structured KB signals with tailored retrieval objectives.

## Supplementary Material

btag490_Supplementary_Data

## Data Availability

Code and data are available at https://github.com/WangXII/edel_repo and https://doi.org/10.5281/zenodo.20368806.
