# Supplementary material for: EDEL: enhancing dense retrievers for curation of biomedical knowledge bases
Source: Bioinformatics. 2026 Jul 2;42(7):btag490. doi: 10.1093/bioinformatics/btag490 (PMC13371770; doi:10.1093/bioinformatics/btag490)
Supplement: btag490_Supplementary_Data [file btag490_supplementary_data.pdf]

Journal Title Here, 2022, 1–4
doi: DOI HERE
Advance Access Publication Date: Day Month Year
Paper

EDEL: Enhancing Dense Retrievers for Curation of Biomedical Knowledge Bases

Xing David Wang[1,†] and Ulf Leser[1,*]

[1]Department of Computer Science, Humboldt-Universität zu Berlin, Unter den Linden 6, 10099 Berlin, Germany

*Corresponding authors: xing.david.wang@hu-berlin.de and leser@hu-berlin.de

FOR PUBLISHER ONLY Received on Date Month Year; revised on Date Month Year; accepted on Date Month Year

Abstract

Motivation: Retrieval of relevant papers from the literature is the first step in curating high-quality biomedical knowledge bases. While BM25 has long been the method of choice, dense retrieval models achieved improved accuracy by embedding queries and documents into dense vector representations. Existing knowledge bases provide a natural source for deriving query-document pairs for training such models. Current training approaches, however, do not take into account that some evidence described by knowledge base entries may only be partially expressed in document abstracts, while the full evidence is often contained in inaccessible full texts, introducing noise into binary relevance labels. In addition, existing approaches only make limited use of the knowledge base structure for selecting negative samples during training.
Results: We propose EDEL, a novel dense bi-encoder for biomedical knowledge base curation to enable curators to find relevant papers for annotation faster. It introduces a loss function using graded relevance scores instead of binary labels to facilitate learning from partially grounded examples, together with a structured sampling strategy that exposes the model to diverse and hard negative examples during training. We evaluate EDEL's performance in two curation settings, namely precision oncology (on CIViC and OncoKB) and post-translational modifications (on UniProt). EDEL outperforms other state-of-the-art models in NDCG@10 by 1.5 and 3.4 percentage points, respectively. Ablation studies show the effectiveness of both innovations. These results indicate that EDEL can substantially improve literature retrieval for biomedical knowledge base curation.
Availability and implementation: Code to reproduce our results is available at: `https://github.com/WangXII/edel_repo`.
Contact: xing.david.wang@hu-berlin.de, leser@hu-berlin.de

A. Dataset

For the PO dataset, we use the CIViC KB[1] (Griffith et al., 2017) and OncoKB[2] (Chakravarty et al., 2017) available upon request using the API. For the UniProt dataset, we use the SwissProt subset of the UniProtKB[3].

We use the same PubMed corpus for both KB retrieval tasks available for download under `https://ftp.ncbi.nlm.nih.gov/pubmed/baseline/`. We include all documents of the 2024 baseline (i.e., published until 19/12/2023) in the corpus which contains around 36 million documents.

We split KB entries across their gene/protein entities while creating train, development and test splits. For instance, all KB entries involving the *KRAS* gene go into the same split, the same for holds true for all other genes such as *MAPK* or *AKT-1*. This prevents a query encountered in the train set from also being evaluated in the test set. Detailed statistics about the train/dev/test split are reported in Tables 1 and 2.

[1] available under `https://civicdb.org/downloads/01-Nov-2022/01-Nov-2022-ClinicalEvidenceSummaries.tsv`
[2] available under `https://www.oncokb.org/api-access`; filter for entries created until 2023/06/07 to reproduce our dataset.
[3] available under `https://ftp.uniprot.org/pub/databases/uniprot/current_release/knowledgebase/complete/uniprot_sprot.dat.gz`

© The Author 2022. Published by Oxford University Press. All rights reserved. For permissions, please e-mail: journals.permissions@oup.com

Table 1. Number of unique gene/protein entities, queries, positive query-document pairs and entity tuples extracted from the precision oncology (PO) KBs CIViC and OncoKB.

	Precision Oncology			
	Train	Dev	Test	All
Genes/Proteins e_1	275	39	81	395
Queries $q = (e_1, ...)$	778	153	133	1,064
Query-answer pairs $(e_1, ..., \mathbf{e_n})$	2,048	354	264	2,666

Table 2. Number of unique gene/protein entities, queries, positive query-document pairs and entity tuples extracted from the UniProt KB for post-translational modifications (PTM).

	PTM			
	Train	Dev	Test	All
Genes/Proteins e_1	1,401	200	401	2,002
Queries $q = (e_1, ...)$	1,732	243	480	2,455
Query-answer pairs $(e_1, ..., \mathbf{e_n})$	2,013	290	558	2,861

Table 3. Positive margin classes on PTM.

		EntitiesInText				
Class	μ	Protein	PTM	Residue	Catalyst	Count
1	0	True	True	True	True	563
2	0.4	Two of three matching			True	1,179
3	0.6	True	True	True	False	1,179
		Two of four matching				1,213
4	0.8	One of four matching				1,294
5	1.0	False	False	False	False	51

B. Abstract string matching and query string construction

We use exact string matching to map knowledge base (KB) entities to their abstract mentions. To improve matching coverage, we use synonym list for the entities, namely NCITheSaurus[4] for drugs and treatments and EntrezGene[5] for genes and UniProtKB for proteins. For variant and residue entities, we fall back to simple synonym expansion rules for matching, e.g., including both alanine 178 and Ala178 as matches for A178.

To construct query strings in the PO dataset, we use official gene symbols from EntrezGene for the gene entities. In the PTM dataset, we include both the official UniProt symbol and its full name in brackets for protein entities.

C. Margin classes on the Post-Translational Modifications (PTM) dataset

In Tables 3 and 4, we report our used margin classes and their corresponding margin value on the PTM dataset.

D. BM25 Negatives

To obtain BM25 negatives for both KB datasets, we retrieved documents that (i) contained at least one synonym of the query gene/protein entity and (ii) did not contain any trigger word associated with the corresponding answer entity type.

For the PO dataset, excluded trigger words included treatment- and clinical-related terms such as *therapy*, *drug*, *clinical trial*, or *chemotherapy*.

For the PTM dataset, excluded trigger words included both general PTM-related terms (e.g., *post-translational modification*, *protein modification*) and PTM-specific lexical variants such as *phosphorylation*, *acetylation*, *methylation*, and related enzyme terms (e.g., *kinase*, *phosphatase*, *acetyltransferase*).

The complete trigger word lists that we filter out for each query are provided in our repository[6].

E. Model training and hyperparameter selection

We fine-tune our EDEL models for 8 epochs, using a learning rate of 2e-5 and a linear warmup schedule for the learning rate taking up 10 percent of the all training steps. We use the same hyperparameters for the fine-tuned MedCPT model and the fine-tuned ColBERTv2 model, except setting the learning rate to 1e-5 for the ColBERTv2 model.

For initial assignments of margin class values, we set the margin for each class as $\mu = 1.0 - \frac{\#Matching}{\#Total}$ where 1.0 denotes the initial margin value for irrelevant pairs (with a cosine distance of 1.0 representing embedding vectors orthogonal to each other), $\#Matching$ denotes the number of matching/overlapping entities in the abstract and KB, respectively, and $\#Total$ denotes the total amount of entity types in the KB curation task. This naturally reduces margin values for complete positives and hard negatives samples while keeping them high for noisy positives and easy negatives. From these initial assignments, we apply grid search to adjust margin values iteratively changing at most margin values for one or two classes at the same time.

We optimize our set of hyperparameters (including the margin values) on the respective development sets. Calculating the full evaluation metrics for each model iteration is not feasible as it requires full indexing of the whole PubMed corpus. Therefore, during model development, we refer to the mean average precision (MAP) metric to evaluate model performance. The MAP is calculated as the mean over the average precision across all development set queries. The average precision for a specific query is given by calculating the precision at every rank k and then averaging it where the candidate documents for each query are made up of the positive sample and its set of training negatives (evaluating on this sample is faster than ranking over whole PubMed). The precision at rank k is defined by the number of relevant documents found in the top-k documents divided by k.

Then, we choose the best performing model according to the MAP metric. As the hyperparameter space is quite extensive, we apply a best-effort search and stop after local convergence of the MAP metric. At the end, we merge classes with the same final margin values together.

In Table 5, we conduct a case study on how sensitive the EDEL model is to different configurations of its margin values and report the results. In the case study, we evaluates how both the addition of a negative margin class as well as the choice of its margin value μ can affect model performance. We augment an EDEL (-LL,-NP,-Class 1 Negatives) ablation by adding back hard negatives from that Class 1. We test three different values for the margin μ: (i) $\mu =$

[4] available under `https://evs.nci.nih.gov/ftp1/NCI_Thesaurus/archive/2022/22.11d_Release/Thesaurus.FLAT.zip`

[5] available under `https://ftp.ncbi.nih.gov/gene/DATA/gene_info.gz`

[6] under `https://github.com/WangXII/edel_repo/blob/master/models/civic_oncokb_retriever.py` and `https://github.com/WangXII/edel_repo/blob/master/po_datasets/uniprot_dictionaries.py`

Table 4. Negative margin classes on PTM.

Class	μ	SameProtein	SamePTM	SameResidue	AnyCatalyst	InText	Count
1	0.4	True	True	False	True	All	2,015
2	0.6	True	One of three overlapping			All	1,260
3	0.8	True	False	False	False	All	5,331
		True	-	-	True	Protein=False	2,425
		False	-	-	True	All	37,980
		BM25 negatives of same protein				-	5,250
4	1.0	True	-	-	False	Protein=False	5,493
		False	-	-	False	All	37,980
5	1.2	Random PubMed negatives				-	37,980

Table 5. Ablation studies by adding negative samples with different margin values on the PTM development set. -LL removes the layered loss function, noisy positives and hard negatives (from Class 2).

**Dataset** (→)	**Post-translational Modifications**					
Development set	NDCG		MAP		Entity Recall	
**Model Name** (↓)	@10	@50	@10	@50	@10	@50
EDEL (-LL,-NP, - Class 1 Neg)	22.74	25.57	18.88	19.44	41.03	52.76
+ Class 1 Neg with $\mu = 0.8$	26.05	28.73	22.41	22.93	39.66	51.03
+ Class 1 Neg with $\mu = 0.4$	20.74	22.90	17.21	17.69	42.07	52.41
+ Class 1 Neg with $\mu = 0.0$	20.17	21.90	16.82	17.14	39.66	51.38

0.8 (same as other negatives), (ii) $\mu = 0.4$ (noisy samples), and (iii) $\mu = 0.0$ (same as positives). As we evaluate on the development set fo the PTM dataset, setting $\mu = 0.8$ interestingly improves MAP and NDCG but reduces Entity Recall, likely underestimating other noisy but relevant documents. In contrast, setting $\mu = 0.4$ or $\mu = 0.0$ degrades performance across all metrics, likely making it harder for the model to discern between the positive and negative documents.

F. Evaluation metrics

Following popular retrieval benchmarks like BEIR (Thakur et al., 2021) and MTEB (Muennighoff et al., 2022), we report the NDCG (normalized discounted cumulative gain) at cutoff threshold $k = 10$ and $k = 50$, that means, considering only the top-k retrieved documents from the answer ranking. NDCG ranges from 0 to 1 with 1 being an perfect ranking having all relevant documents in the first search results. Thus, it quantifies how well our retrieval systems can identify the annotated gold document of a given KB entry and its respective query. NDCG is defined as follows:

$$\text{NDCG@}k = \frac{\text{DCG@}k}{\text{IDCG@}k}$$

where

$$\text{DCG@}k = \sum_{i=1}^{k} \frac{rel_i}{\log_2(i+1)}$$

and rel_i is the relevance score $\in \{0, 1\}$ of the item at rank i, with 0 denoting irrelevance and 1 relevance. The ideal DCG (IDCG@k) is computed by sorting the ground truth relevance scores in descending order and calculating DCG on this ideal ranking.

G. Task Difficulty

Table 6. NDCG@10 scores for the baselines on the newly introduced datasets Precision Oncology and Post-Translational Modifications (PTM) and the BioASQ dataset as reported in (Thakur et al., 2021).

**Model Name**	**Precision Oncology**	**PTM**	**BioASQ**
BM25	11.1	20.5	46.5
ColBERTv2	10.9	18.5	47.4
MedCPT	9.1	13.1	33.2

Both new KB curation tasks prove to be challenging retrieval problems: When we compare baseline performances on our new KB curation retrieval tasks to the BioASQ (Tsatsaronis et al., 2015) task (that consists of question-answer pairs over the whole biomedical domain to be answered via PubMed abstracts), none of our three baseline models exceeds 15 percent in NDCG@10 on the PO task and 25 percent on the PTM task. For comparison, the same retriever models achieve NDCG@10 scores of up to 50 percent (Thakur et al., 2021) on BioASQ (see Table 6).

H. Sample Retrieval

In Table 7, we present some sample queries and their documents retrieved by EDEL, focusing particularly on documents which have not been annotated as gold by the annotators during the retrieval process.

I. Fine-tuning MedCPT

Similarly to ColBERTv2 Santhanam et al. (2022), we also fine-tuned MedCPT (Jin et al., 2023) twice, once on complete positives only, once including noisy positives as well. In Table 8, we report the corresponding results. Performance for both approaches are pretty comparable on the PO dataset whereas training on noisy positives results in much worse retrieval performance on the PTM dataset, confirming the observations of model drift due to added noise as observed in the ColBERTv2 dataset.

References

D. Chakravarty, J. Gao, S. Phillips, R. Kundra, and H. e. a. Zhang. OncoKB: A Precision Oncology Knowledge Base. *JCO Precision Oncology*, (1):1–16, Nov. 2017.

M. Griffith, N. C. Spies, K. Krysiak, J. F. McMichael, and A. C. e. a. Coffman. CIViC is a community knowledgebase for expert crowdsourcing the clinical interpretation of variants in cancer. *Nature Genetics*, 49(2):170–174, Feb. 2017.

Q. Jin, W. Kim, Q. Chen, D. C. Comeau, L. Yeganova, W. J. Wilbur, and Z. Lu. MedCPT: Contrastive Pre-trained

Table 7. Sample queries and retrieved PubMed documents by EDEL. Key entities are marked in italics. The first three samples show instances of successful retrieval with all entities matching in the retrieved abstract (gene, variant and treatment) whereas the last sample warrants further reading into the full text as the specific variant is not mentioned in the abstract.

Query	Document	Rank	Gold document
1. Treatment for gene PTPRT and promoter hypermethylation?	31316618: ... We further show that *PTPRT promoter methylation* is significantly associated with ... responsiveness to *STAT3 inhibitors* in clinical development ...	11	Yes
2. Treatment for gene FGFR1 and variant ZNF198::FGFR1?	17698633: ... The viability of Ba/F3 cells transformed to IL3 independence by *ZNF198-FGFR1* or BCR-FGFR1 was specifically inhibited by *TKI258*...	2	No
3. Treatment for gene CDKN2A and variant Deletion?	34592265: ... revealed that *deletion of cyclin dependent kinase inhibitor 2A (CDKN2A)* was highly associated with primary resistance to PD-1 blockade ...	2	No
4. Treatment for gene ERCC2 and variant K751Q?	30290956: ... *ERCC2 mutations* predicted to result in increased cisplatin sensitivity were enriched in primary versus secondary MIBC ...	2	No

Table 8. Performances on the datasets Precision Oncology and Post-translational modifications measured by NDCG, MAP and Entity Recall @10 and @50 in %. n denotes the number of queries. Best score in each column is marked in bold.

**Dataset** (→)	**Precision Oncology**						**Post-translational Modifications**					
	NDCG		MAP		Entity Recall		NDCG		MAP		Entity Recall	
**Model Name** (↓)	@10	@50	@10	@50	@10	@50	@10	@50	@10	@50	@10	@50
MedCPT	12.91	15.46	10.87	11.34	28.41	53.79	19.64	23.23	15.73	16.54	40.14	57.53
MedCPT (+NP)	12.68	14.99	10.97	11.46	30.68	53.79	13.20	15.56	10.97	11.45	29.57	48.75

Transformers with large-scale PubMed search logs for zero-shot biomedical information retrieval. *Bioinformatics*, 39(11): btad651, Nov. 2023.

N. Muennighoff, N. Tazi, L. Magne, and N. Reimers. Mteb: Massive text embedding benchmark. *arXiv preprint arXiv:2210.07316*, 2022.

K. Santhanam, O. Khattab, J. Saad-Falcon, C. Potts, and M. Zaharia. ColBERTv2: Effective and Efficient Retrieval via Lightweight Late Interaction. In *Proceedings of the 2022 Conference of the North American Chapter of the Association for Computational Linguistics: Human Language Technologies*, pages 3715–3734, Seattle, United States, July 2022. Association for Computational Linguistics.

N. Thakur, N. Reimers, A. Rücklé, A. Srivastava, and I. Gurevych. Beir: A heterogenous benchmark for zero-shot evaluation of information retrieval models. *arXiv preprint arXiv:2104.08663*, 2021.

G. Tsatsaronis, G. Balikas, P. Malakasiotis, I. Partalas, M. Zschunke, M. R. Alvers, D. Weissenborn, A. Krithara, S. Petridis, D. Polychronopoulos, et al. An overview of the bioasq large-scale biomedical semantic indexing and question answering competition. *BMC bioinformatics*, 16:1–28, 2015.
